# A Transgenerational Endocrine Signaling Pathway in Crustacea

**DOI:** 10.1371/journal.pone.0061715

**Published:** 2013-04-17

**Authors:** Gerald A. LeBlanc, Ying H. Wang, Charisse N. Holmes, Gwijun Kwon, Elizabeth K. Medlock

**Affiliations:** Department of Environmental and Molecular Toxicology, North Carolina State University, Raleigh, North Carolina, United States of America; Baylor College of Medicine, United States of America

## Abstract

**Background:**

Environmental signals to maternal organisms can result in developmental alterations in progeny. One such example is environmental sex determination in Branchiopod crustaceans. We previously demonstrated that the hormone methyl farnesoate could orchestrate environmental sex determination in the early embryo to the male phenotype. Presently, we identify a transcription factor that is activated by methyl farnesoate and explore the extent and significance of this transgenerational signaling pathway.

**Methodology/Principal Findings:**

Several candidate transcription factors were cloned from the water flea *Daphnia pulex* and evaluated for activation by methyl farnesoate. One of the factors evaluated, the complex of two bHLH-PAS proteins, dappuMet and SRC, activated a reporter gene in response to methyl farnesoate. Several juvenoid compounds were definitively evaluated for their ability to activate this receptor complex (methyl farnesoate receptor, MfR) in vitro and stimulate male sex determination in vivo. Potency to activate the MfR correlated to potency to stimulate male sex determination of offspring (pyriproxyfen>methyl farnesoate>methoprene, kinoprene). Daphnids were exposed to concentrations of pyriproxyfen and physiologic responses determined over multiple generations. Survivial, growth, and sex of maternal organisms were not affected by pyriproxyfen exposure. Sex ratio among offspring (generation 2) were increasingly skewed in favor of males with increasing pyriproxyfen concentration; while, the number of offspring per brood was progressively reduced. Female generation 2 daphnids were reared to reproductive maturity in the absence of pyriproxyfen. Sex ratios of offspring (generation 3) were not affected in this pyriproxyfen lineage, however, the number of offspring per brood, again, was significantly reduced.

**Conclusions:**

Results reveal likely components to a hormone/receptor signaling pathway in a crustacean that orchestrates transgenerational modifications to important population metrics (sex ratios, fecundity of females). A model is provided that describes how these signaling processes can facilitate population sustainability under normal conditions or threaten sustainability when perturbed by environmental chemicals.

## Introduction

Hormones in the fetal environment regulate a variety of processes that orchestrate physiologic function in the resulting offspring. For example, intrauterine fetal position of mice, with respect to the sex of its adjacent litter mates and thus the hormonal environment of the fetus, influences later events such as the timing of puberty and sexual behavior [1]. Perturbations in the prenatal hormonal milieu can result in inter-individual variability in the expression of these programmed traits as well as disease [2]. Indeed, administration of hormones or hormone mimics to maternal rodents has resulted in the production of offspring with increased susceptibility to prostate cancer [3], mammary tumors [4], obesity [5], and glucose intolerance [5].

Changes in fetal programming due to alterations in the hormonal environment of the developing fetus, be it from maternal influences, *in utero* sibling influences, or maternal exposure to environmental chemicals and drugs, are generally considered to be caused by disruptions or alterations in hormonal regulation of epigenetic programming events. Various components of the epigenetic machinery are under the control of hormones and fetal exposure to hormones or their mimics have been shown to alter epigenetic modifications of several genes [6]. However, a precise understanding of the linkage between endocrinology and fetal programming is lacking.

Environmental sex determination provides a plausible phenomenon that could serve well to define the mechanistic linkages between endocrinology and fetal programming. Environmental sex determination is the ubiquitous process among metazoans whereby sex is determined, not by sex chromosomes allocated to the fetus by its parents, but by environmental influences on the maternal organism or fetus. Environmental factors responsible for sex determination of offspring include temperature [7], nutrition [8], photoperiod [9], and population density [10]. Environmental sex determination serves to provide population sex ratios that will maximize sustainability of the population under incipient environmental conditions [11]. Generally, the environmental cue is considered to stimulate the release of a chemical signaling molecule (i.e., hormone) that orchestrates the sex programming of the neonate [12]. Despite the ubiquity with which environmental sex determination occurs, the process itself remains poorly understood.

Branchiopod crustaceans, such as *Daphnia* sp., are cyclic parthenogens that are subject to environmental sex determination [13]. Under suitable environmental conditions, daphnid populations consist largely of females that reproduce asexually. This clonal reproduction provides for the rapid expansion of the population. However, in response to specific environmental cues, that typically represent a limiting factor to unregulated population growth, the daphnids will produce male offspring. Male sex determination is under endocrine control. The sequiterpenoid hormone methyl farnesoate programs oocytes in late stages of maturation to develop into male offspring [14], [15]. The males mate with sexually receptive females producing embryos that are more genetically diverse and less likely to carry gene mutations [16]. These embryos are typically in a diapause state and can develop once in a different time or space that is more conducive to parthenogenetic population expansion.

Daphnids can serve as an ideal model for the evaluation of transgenerational signaling owing to: a) environmental sex determination in this taxa is highly suitable to mechanistically evaluate transgenerational signaling; b) populations can be readily reared and offspring sex can be controlled in the laboratory [14], [17]; and c) the genome of a member of this taxa (*Daphnia pulex*) has been fully sequenced [18]. In the present study, we sought to identify the endocrine-related transcription factors that translate environmental signals received by the mother to sex determination of her offspring. Three transcription factors were characterized and evaluated for involvement in environmental sex determination. DappuPNR and dappuDSF are member of the NR2E group of nuclear receptors [19]. Members of this group of nuclear receptors are important in various aspects of neural development including sexual orientation sex-specific and reproductive behavior [20], [21]. Thus, members of this group of transcription factors in daphnids were considered as candidates for contributing to environmental sex determination. The methoprene-tolerant (Met) protein is a member of the bHLH-PAS family of transcription factors and is a component of the juvenoid hormone signaling pathway in insects [22]. Consequently, we considered this protein to be a candidate for mediating the action of methyl farnesoate, the unepoxidated form of juvenile hormone III, in crustaceans. We also explored the significance of this transgenerational signaling pathway with respect to population sustainability parameters.

## Results

### Transcription Factor Cloning

**Figure 1:**
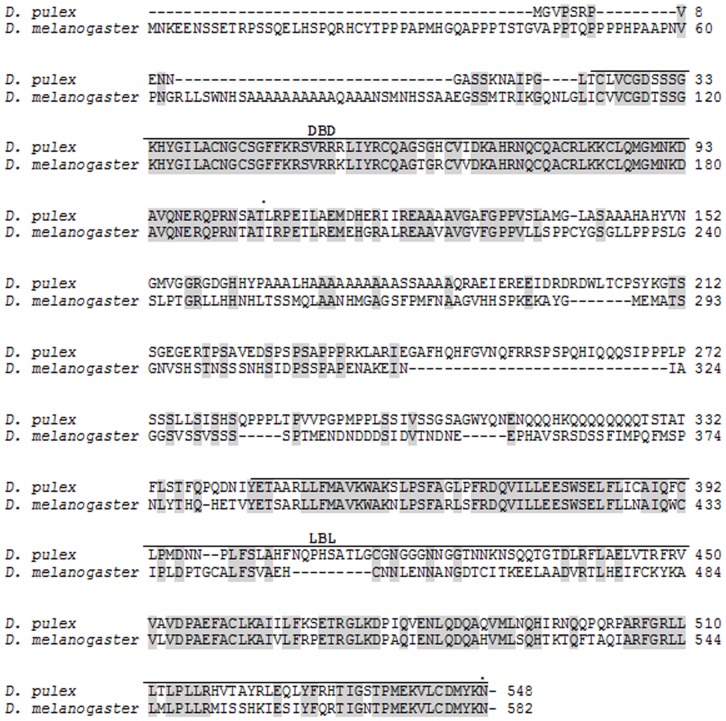
Amino acid sequence of *D. pulex* PNR deduced from the nucleotide sequence of dappuPNR (Fig. S1) and aligned to PNR from *D. melanogaster.* The *D. melanogaster* sequence was deduced from the nucleotide sequence provided in GeneBank (accession # NP_611032.2). The DNA-binding domain (DBD) and the ligand-binding domain (LBD) are indicated. Common amino acids between the two sequences are shaded.

**Figure 2:**
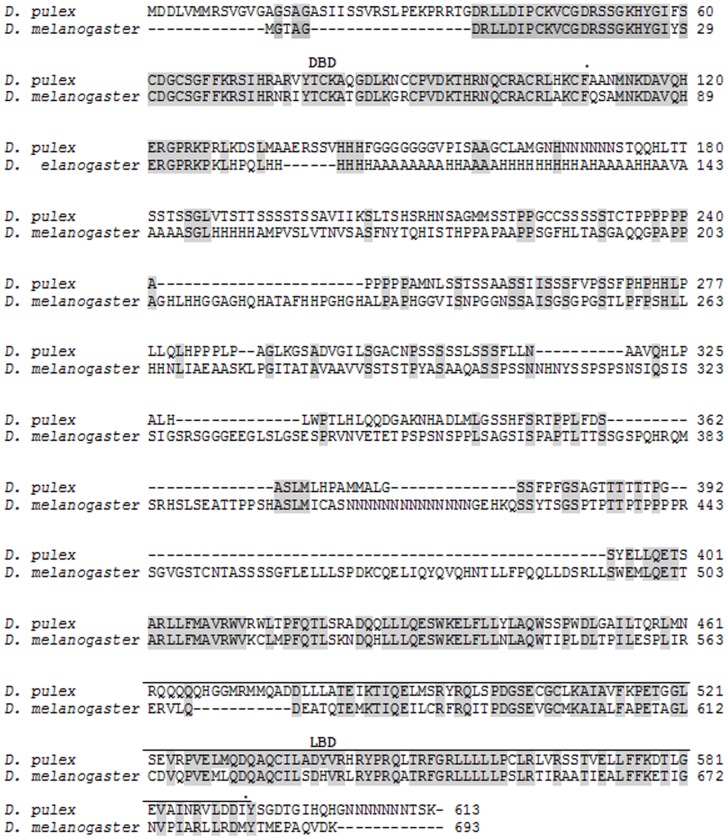
Amino acid sequence of *D. pulex* DSF deduced from the nucleotide sequence of dappuDSF (Fig. S2) and aligned to DSF from *D. melanogaster.* The *D. melanogaster* sequence was deduced from the nucleotide sequence at Gene Bank (accession number AAD05225.1). The DNA-binding domain (DBD) and the ligand-binding domain (LBD) are indicated. Common amino acids between the two sequences are shaded.

**Figure 3:**
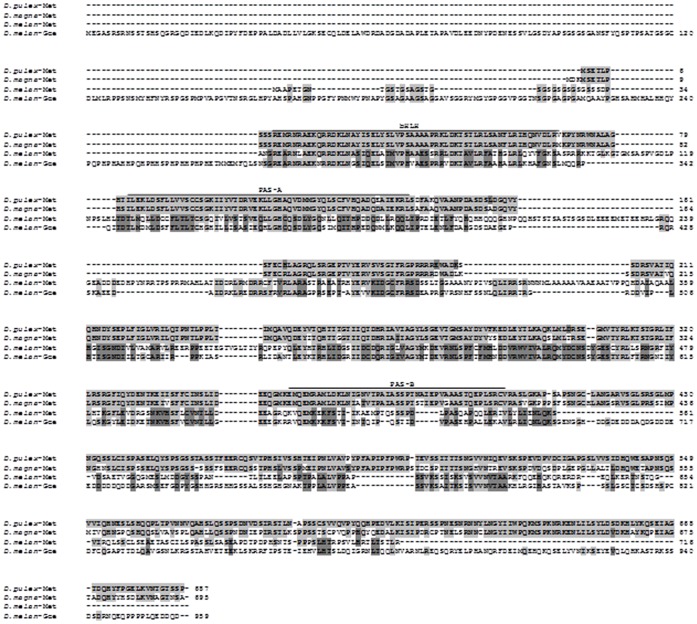
Aligned amino acid sequences of *D. magna* and *D. pulex* Met deduced from the nucleotide sequences of dapmagMet and dappuMet (Figs. S3 and S4, respectively) and aligned to Met and Gce from *D. melanogaster*. The *D. melanogaster* sequences were deduced from the nucleotide sequence at GeneBank (accession numbers NM_078571 and NP_001259566.1). The bHLH and PAS domains (A and B) are indicated. Identical amino acids are indicated by the same shading.

The transcription factors dappuPNR, dappuDSF, and dappuMet were cloned from *D. pulex* using the deduced gene sequences derived from the published sequenced genome of the organism (wFleaBase.org) [18], [19]. Nucleotide sequences of the cloned genes (cDNAs) are presented in the Supporting Information (Figs. S1, S2, and S3). Deduced amino acid sequences for the gene products are provided in Figs. 1, 2, and 3. The dappuPNR gene product was 548 amino acids in length and contained DNA-binding and ligand-binding sites characteristic of most other members of the nuclear receptor family. Its DNA-binding site was 89% identical and its ligand-binding site was 61% identical to those of PNR from *Drosophila melanogaster*. The dappuDSF gene product was 613 amino acids in length and also contained DNA-binding and ligand-binding sites. Its DNA-binding site was 90% identical and its ligand-binding site was 66% identical to those of DSF of *D. melanogaster*.

The Met cDNA was cloned from both *D. pulex* (dappuMet; Fig. S3) and *D. magna* (dapmagMet; Fig. S4) since *D. magna* was used for subsequent whole animal experiments and Met proved to be most relevant to these experiments. The sequenced dappuMet cDNA was highly similar to the sequence derived from wFleaBase. Overall, the two sequences were 97% identical with 100%, 91%, and 98% identity within the bHLH, PAS-A, and PAS-B domains, respectively. The major difference between the two sequences was an additional stretch of 10 nucleotides in the sequenced cDNA just 3′ of the bHLH domain which may have been lost in the wFleaBase sequence due to an error in intro/exon designations. The sequenced dappuMet and dapmagMet cDNAs were also highly similar with 100%, 98%, and 88% identity in the bHLH, PAS-A, and PAS-B domains, respectively (Fig. 3). The bHLH domain is typically involved in protein dimerization and, in some cases, DNA binding [23]. The PAS domains are typically involved in dimerization to partner transcription factors or in binding, as a co-activator, to transcription factors, depending upon the specific function of the bHLH-PAS protein [23]. No evidence of dappuMet paralogs was discerned during the cloning of the dappuMet cDNA.

The sequenced dappuMet was 64%, 36%, and 26% identical to the bHLH, PAS-A, and PAS-B domains of the *Drosophila melanogaster* Met, respectively (Fig. 3). In contrast, these domains were 62%, 24%, and 21% similar to the respective domains of the *D. melanogaster* Gce, a paralog of Met (Fig. 3). Taken together, the evidence supports the identification of the sequenced cDNAs from *D. pulex* and *D. magna* as being Met and not a Met paralog. Results also support the use of *D. magna* as a surrogate to *D. pulex* in subsequent whole animal experimentation.

### Activation of the Transcription Factors by Methyl Farnesoate

**Figure 4:**
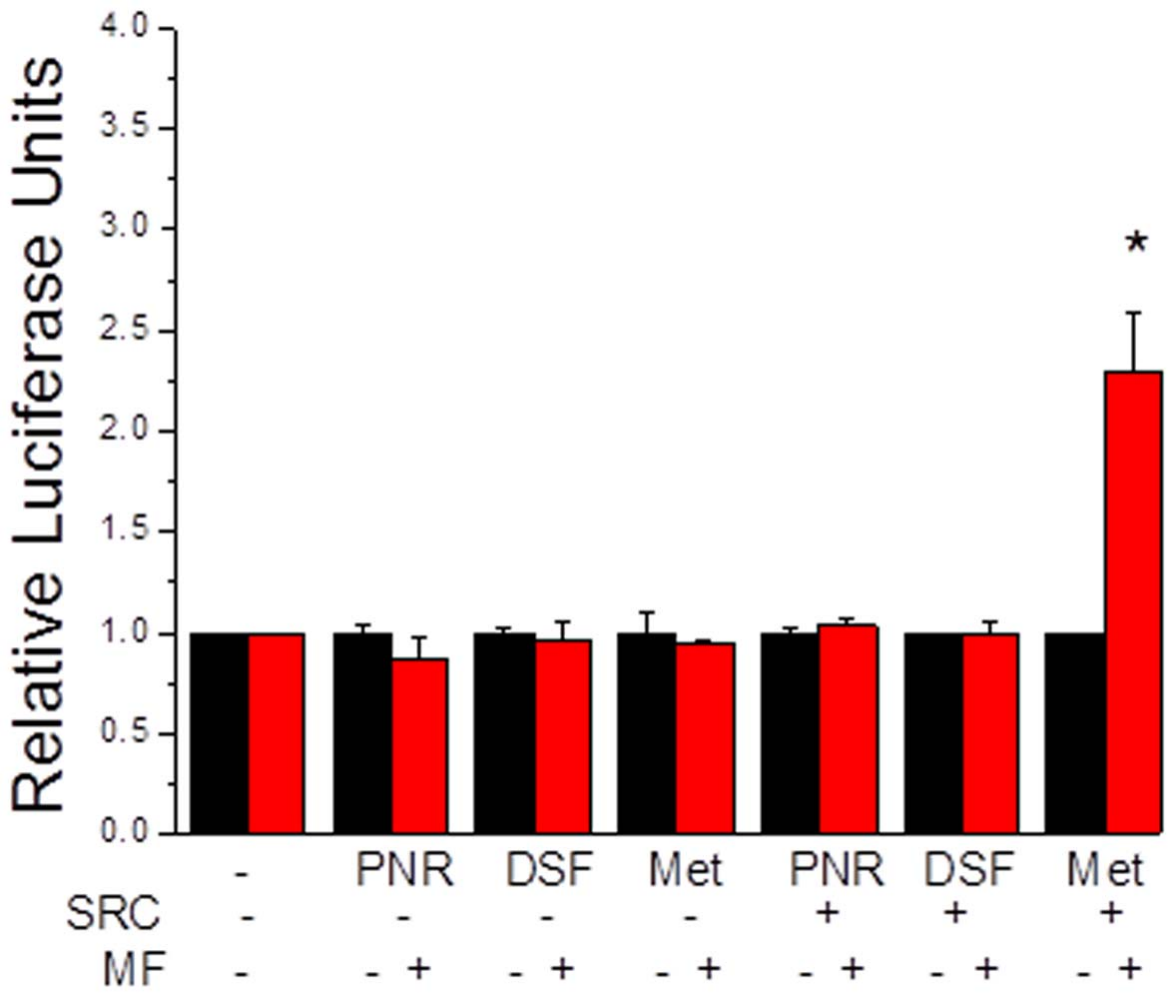
Activation of a GAL4-driven luciferase reporter gene by dappuPNR-GAL4, dappuDSF-GAL4, and dappuMet-GAL4 in the presence and absence of SRC (1 µg plasmid DNA transfected) and methyl farnesoate (MF, 10 µM). An asterisk denotes a significant difference (p<0.05) from the respective assay performed in the absence of MF. All data are represented by the mean and standard deviation of three replicate assays.

**Figure 5:**
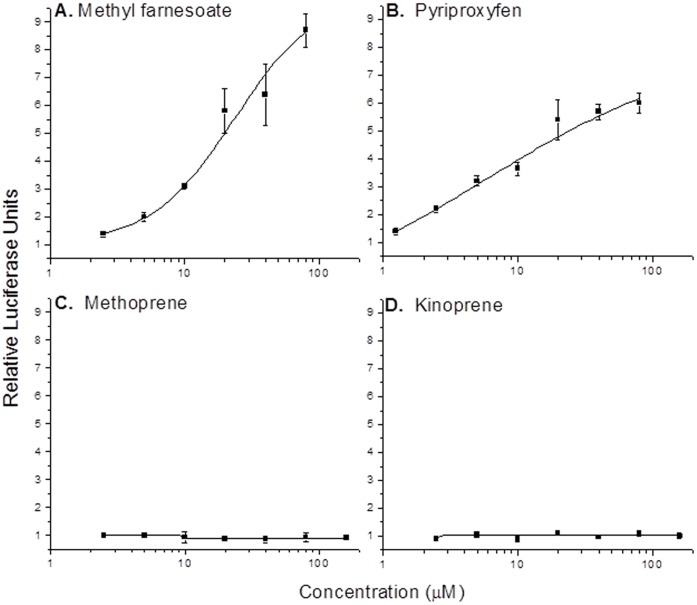
Activation of a GAL4-driven luciferase reporter gene by the dappuMfR (Met-GAL4:SRC) by different concentrations of putative ligands. Data represents the mean (data point) and standard deviation (error bars) of three replicate assays.

Constructs of the transcription factors containing the Gal4 DNA binding domain were used in transcription reporter assays where luciferase was the reporter gene which contained GAL4 binding sites upstream of the transcription start site. In the initial screen, none of the transcription factors stimulated luciferase expression either alone or in the presence of 10 µM methyl farnesoate (Fig. 4). SRC is a bHLH-PAS protein that is known to associate with a number of nuclear receptor family of proteins, as well as, bHLH-PAS transcription factors [24]. We therefore, co-transfected insect SRC (previously identified as mosquito-FISC [25]) into the transfection reporter assays and evaluated methyl farnesoate responsiveness. SRC had no effect in reporter assays involving dappuPNR and dappuDSF (Fig. 4). However, dappuMet did activate gene transcription in response to methyl farnesoate when SRC was added to the assay (Fig. 4). Concentration-response analyses revealed that methyl farnesoate activated the dappuMet –SRC complex, hereafter referred to as the methyl farneosate receptor (MfR), with maximum activation of ∼9-fold with a potency (EC_50_) of 16 µM (Fig. 5A).

Three compounds that function as juvenile hormone mimics in insects were selected to determine whether these compounds also activated the MfR. Of the three compounds selected, only pyriproxyfen activated the MfR (Fig. 5B). Maximum activation of the complex was ∼2/3 of that observed with methyl farnesoate though this compound appeared more potent with an estimated EC_50_ of 4.8 µM (Fig. 5B). Neither methoprene nor kinoprene activated the MfR at concentrations as high as 120 µM (Figs. 5C,D).

### Male Sex Determination

**Figure 6:**
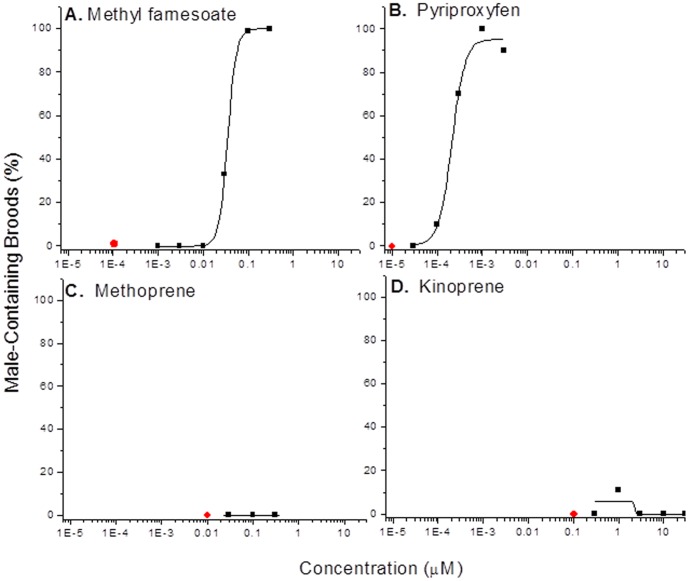
Percentage maternal daphnids (*D. magna*; n = 10) that produced male-containing broods following exposure to putative MfR ligands. Red dots denote the percentage male-containing broods among 10 daphnids that were not exposed to ligands (negative control).

We have shown that methyl farnesoate is a male sex determinant in daphnids [14]. Experiments next were performed to determine whether the relative potency of methyl farnesoate and the juvenile hormone mimics correlated to the relative potency of these compounds to activate the MfR. Both methyl farnesoate and pyriproxyfen stimulate male sex determination among offspring of exposed maternal organisms (Fig. 6A,B) with pyriproxyfen being more potent. EC_50_ values for male offspring production were 34 nM and 0.22 nM for methyl farnesoate and pyriproxyfen, respectively. Neither, methoprene nor kinoprene stimulated male offspring production at the maternal exposure concentrations tested which were limited by toxicity (methoprene) or solubility (kinoprene) (Fig. 6C,D). The potency ranking of the four compounds were comparable with respect to the activation of the MfR and male sex determination. Although, the magnitude of difference between methyl farnesoate and pyriproxyfen was much greater for male sex determination as compared to activation of the MfR.

### Transgenerational Impacts on Life History Parameters

**Figure 7:**
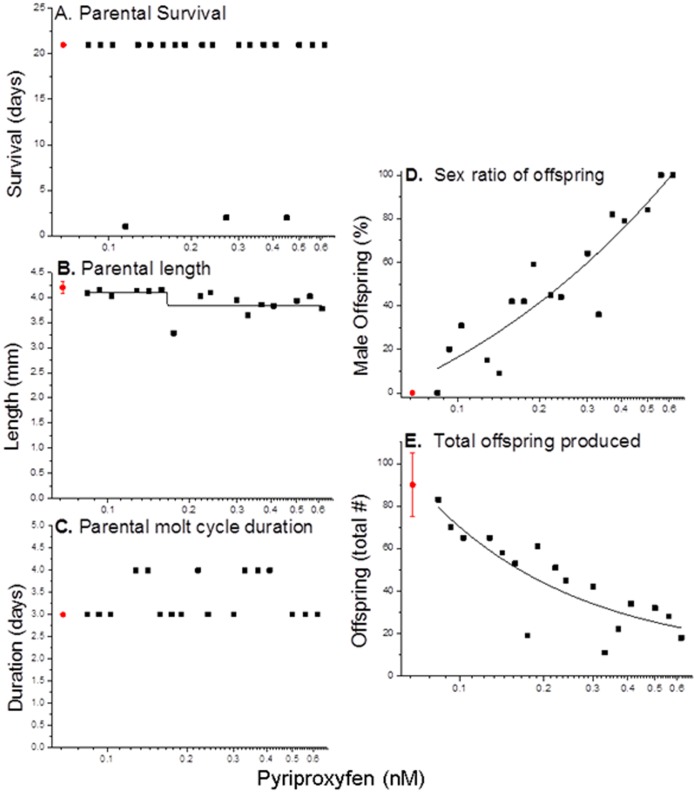
Physiological responses of daphnids (*D. magna*) exposed to concentrations of the MfR ligand pyriproxyfen through their life cycle. Each black data point represents the response of a single daphnid. Red dots depict the performance (mean±standard deviation) of ten unexposed daphnids.

Having demonstrated that pyriproxyfen was most potent in activating the MfR we next evaluated whether elevated levels of the MfR ligand in the maternal organisms (generation 1) elicited responses specifically in offspring (generation 2) or next generation offspring (generation 3). Continuous exposure of first generation organisms to concentration of pyriproxyfen ranging from 0.084 to 0.62 nM had no discernible effect on longevity (Fig. 7A), growth (Fig. 7B) or molt frequency (Fig. 7C). All individuals exposed to pyriproxyfen, as well as controls, matured as reproductively competent females. However, male:female sex ratios of offspring (generation 2) increased with increasing concentration of pyriproxyfen and ranged from all female offspring at the exposure concentration of 0.084 nM pyriproxyfen to all male offspring at 0.56 nM pyriproxyfen (Fig. 7D). The magnitude of this effect was comparable to that observed in previous experiments (Fig. 6B) indicating that the effect of pyriproyfen was not cumulative over the duration of exposure but rather reflected the magnitude of exposure as it occurred during a selected window of susceptibility of the prenatal second generation organisms. Further, the number of second generation individuals within a brood decreased with increasing concentration of pyroproxyfen (Fig. 7E) suggesting that pyriproxyfen decreased the number of oocytes recruited for maturation or increased the number of oocytes/embryos lost during the maturation process. Thus, pyriproxyfen had no discernible effect on parental organisms while modifying the development of neonates.

**Figure 8:**
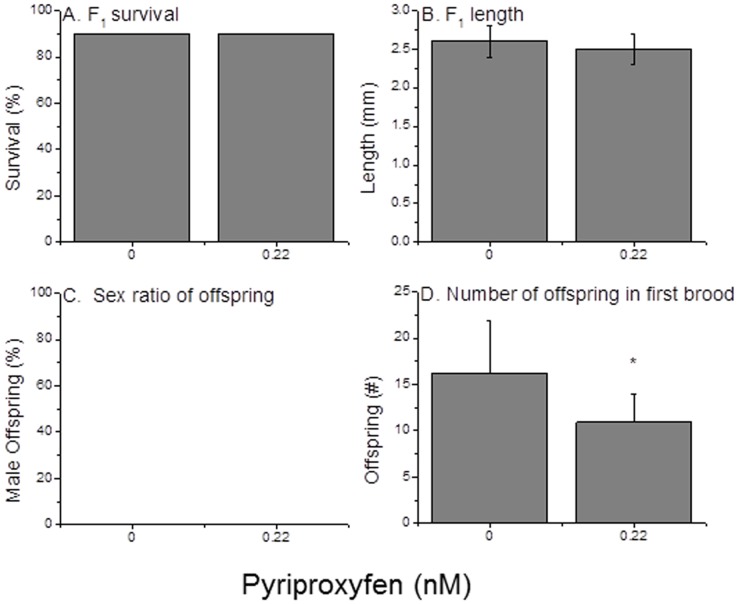
Phsiologic performance of daphnids (*D. magna*), produced by maternal organisms that were exposed to either 0.00 or 0.22 nM pyriproxyfen. These offspring were reared in the absence of pyriproxfen. Data represent the mean and standard deviation (where appropriate) of ten individuals. An asterisk denotes a significant (p<0.05) difference between the treatments.

One female second generation neonate derived from each of ten first generation organisms exposed to 0.22 nM pyriproxyfen was isolated and reared to maturity in the absence of pyriproxyfen. These second generation female neonates all were derived from broods that contained both male and female offspring. Thus, even female offspring were likely exposed to a near sex-determining concentration of pyriproxyfen during prenatal development. Ten control neonates were similarly isolated and reared. There were no significant differences in survival and growth between the second generation pyriproxyfen-exposed lineage and the control daphnids (Fig. 8A, B). Furthermore, all offspring produced (third generation daphnids) in this experiment were female (Fig. 8C). However, consistent with reduced brood sizes observed among pyriproxyfen-exposed daphnids in the previous generation, broods of third generation organisms produced by the pyriproxyfen-exposed lineage were significantly smaller than broods produced by control daphnids (Fig. 8D).

## Discussion

It has been recognized for decades that the hormone methyl farnesoate plays many important roles in crustacean development and reproduction [26]. Yet the receptor protein that mediates the activity of methyl farnesoate has remained an enigma. The close structural and function identity of methyl farnesoate to the insect hormone JHIII has led to speculation that these two hormones may function through some signaling pathway common to insects and crustaceans [27]. Ultraspiracle, the retinoid X receptor ortholog in *D. melanogaster,* was hypothesized to be the functional target of JHIII binding in this insect species [28]. However, we found no evidence to suggest that daphnid RXR is activated by methyl farnesoate [29], [30]. Although, methyl farnesoate did appear to bind to daphnid RXR resulting in synergistic activation of the daphnid ecdysteroid receptor complex (EcR:RXR) by 20-hydroxyecdysone [29]. Recently, we identified the nuclear receptors PNR and DSF within the *D. pulex* genome [19] and presently, we cloned the respective cDNAs. Both nuclear receptors were viewed as candidate methyl farnesoate receptors as members of this nuclear receptor group (NR2E) contribute to sexually dimorphic development in insects [31]. Neither receptor was activated by methyl farnesoate in the reporter gene assay.

We also cloned the methoprene tolerant (Met) gene ortholog from *D. pulex and D. magna.* This bHLH-PAS protein was recently shown to be a strong candidate as a JHIII-dependent transcription factor in mosquito [25]. Daphnid Met alone was unable to activate the reporter gene in the presence of methyl farnesoate. However, when co-transfected with SRC derived from mosquito [25], a functional methyl farnesoate-dependent activator of gene transcription was created. We refer to this receptor complex (Met-SRC) as the methyl farneosoate receptor (MfR). Efforts to clone and express the daphnid SRC are underway, but has proven challenging due to the large size of the gene (>7600 bp). Presently, it is not known whether SRC functions as a partner transcription factor to Met (i.e., contributes to DNA binding) or functions as a non-DNA binding coactivator. It is highly improbable that SRC was independently responsible for reporter gene activation since it did not possess the GAL4 DNA-binding domain. Furthermore, the presence of SRC in experiments involving PNR or DSF did not result in reporter gene activation.Previously, we demonstrated that methyl farnesoate is a male sex determinant in daphnids (*D. magna*) [14]. Subsequently, we and others have shown that methyl farnesoate functions as a sex determinant in other Cladoceran species and some insecticidal juvenile hormone mimics are capable of mimicking this action of methyl farnesaote [15], [17], [32], [33]. Having now identified a candidate MfR in daphnids, we evaluated whether the potency of putative ligands of the MfR correlated to their ability to stimulate male sex determination. The insecticide pyriproxyfen was a potent activator of the MfR and was extremely potent at stimulating male sex determination in vivo. Pyriproxyfen was approximately 3-times as potent as methyl farnesoate in activating the MfR in the mid-range of the concentration-response curve. However, the insecticide was approximately 150-times more potent in stimulating male sex determination. This increased potency in vivo may be due to differences in in vivo-relevant pharmacokinetic parameters such as uptake, distribution, metabolism, and elimination between the two ligands.

The JHIII mimics, methoprene and kinoprene, were unable to activate the MfR and also were inactive as male sex determinants in vivo. Methoprene was previously shown to have weak activity as a male sex determinant [34]. This subtle difference in response between studies may reflect strain differences in the MfR or differences in the manufacturers lots of methoprene used. Regardless, potent activators of the MfR (methyl farnesaote and pyriproxyfen) were shown to be potent male sex determinants in vivo; while, JHIII mimics that were inactive with the MfR also were unable to stimulate the production of male offspring in vivo. These observations support the hypothesis that MfR activation by methyl farnesoate is responsible for male sex determination in daphnids. Additional studies of MfR-ligand, MfR-protein, and MfR-DNA interactions are warranted to definitively establish this putative mechanistic linkage between MfR activation by methyl farnesoate and male-sex determination.

**Figure 9:**
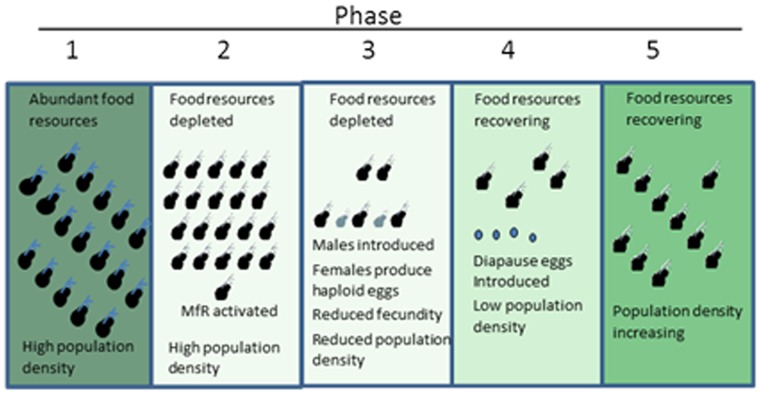
Proposed transgenerational population consequences of activation of the MfR resulting from depleted food resources and high population density.

Experiments on the physiologic responses of daphnids to the potent MfR ligand pyriproxyfen demonstrated the profound multigenerational consequences of activation of this hormonal pathway. Though pyriproxyfen produced no discernible effects on the endpoints measured among parental (generation 1) organisms, these organisms produced progressively more male offspring (generation 2) with increasing exposure concentration of the hormone mimic. Further, female offspring (generation 2) derived from a pyriproxyfen-exposed lineage but whose only potential for exposure to pyriproxyfen was early in development produced fewer offspring (generation 3) than organisms derived from an unexposed lineage. These effects provide novel insight into the manner in which methyl farnesoate may regulate daphnid populations through multiple generations (Fig. 9). Under conditions of food abundance, daphnids reproduce asexually with maternal organisms producing large broods of all-female offspring. These offspring mature and continue the asexual reproductive cycle resulting in rapid population growth (Fig. 9, Phase 1). Ultimately, food resources are depleted and population density is very high (Fig. 9, Phase 2). These duel conditions cause an elevation in methyl farnesoate in maternal organisms resulting in activation of the MfR and the production of male offspring and a reduction in the rate of offspring production (Fig. 9, Phase 3). Population density declines, the population now has viable males, and through presently unidentified stimuli, females produce haploid eggs and become sexually receptive. The population density continues to decline due to the transgenerational suppression of fecundity by the original activation of the methyl farnesoate signaling pathway and fertilized diapause embryos (resting eggs) are introduced into the population (Fig. 9, Phase 4). The reduced density of feeding organisms allows for recovery of food resources, diapause eggs hatch, and the asexual population growth cycle is restored (Fig. 9, Phase 5). A significant data gap in this hypothesis is the present lack of demonstration that methyl farnesoate levels are elevated in daphnids in response to food restriction and high population density (which are known to stimulate the production of male offspring in *D. magna* [10]).

**Figure 10:**
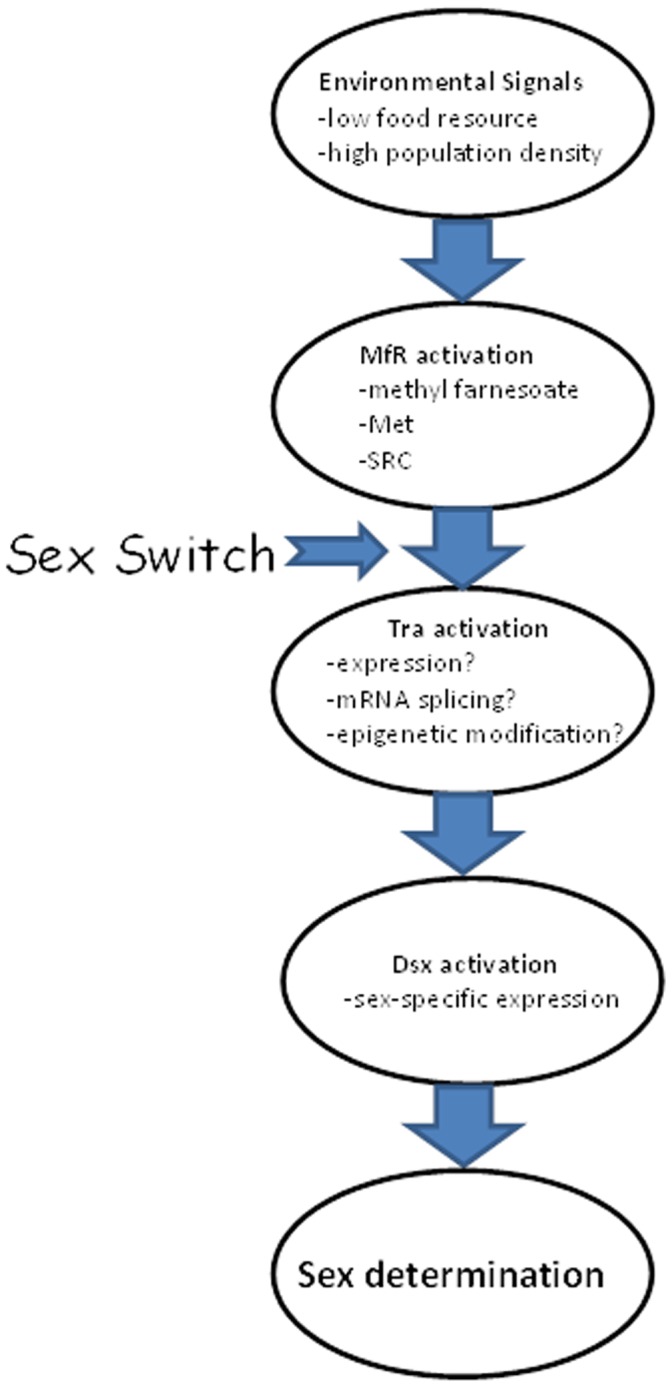
Proposed mechanistic linkage whereby environmental signals receive by material organisms results is sex determination of next generation individuals.

Recently generated information on the molecular contributors to the sex determining pathway of Cladocera provides for assembly of a credible chain of events that link the initiating event (environmental signals) to the apical event (male sex determination) (Fig. 10). We had previously demonstrated that low food resources coupled with high population density are the initiating environmental signals for male sex determination in *D. magna* [10]. We also were the first to demonstrate that the crustacean hormone methyl farnesoate programs maturing oocytes to develop into males [14]. Presently, we show that the the Met:SRC complex (MfR) provides a functional target for mediating the activity of methyl farnesoate. The *transformer* gene (Tra*)* has been identified as the initial determinant of sex differentiation in several insect species [35]. The Tra gene has been identified in *D. magna* but its functionality in the sex-determining pathway is yet to be determined [36]. We propose that methyl farnesoate-activated MfR orchestrates a sex-specific modification to Tra that dictates downstream events leading to male or female differentiation. Essentially, we propose that the default sex in daphnids is female, but activated MfR triggers a “sex switch” that initiates a trajectory for Tra towards male sex differentiation. In insects, the *doublesex* gene (Dsx) is the target of Tra [37]. Dsx protein then orchestrates male or female sex differentiation [38]. In *D. magna*, Dsx expression during early embryogenesis also is responsible for male sex differentiation [39]. Thus, the sex switch may involve the induction of doublesex expression by Tra. A major gap in this proposed pathway is the lack of functional characterization of Tra in Cladocerans.

Results of the present study not only help to elucidate the molecular signaling pathway that links environmental stimuli to sex differentiation, but provide insight into how environmental chemicals can disrupt such signaling pathways resulting in profound transgenerational consequences. Here, we demonstrate that exposure of maternal daphnids to extremely low (parts per trillion) concentrations of an insecticide could dramatically alter sex ratios in the subsequent generation and compromise fecundity of reproductively competent females for at least two generations. Short term reductions in population size of this important food source for juvenile fishes would likely occur under this scenario. Although, long-term consequences are questionable due to the ability of daphnid populations to rapidly recover [40]. None the less, the scenario described herein provides a model that depicts why concern exists for the presence of endocrine disrupting chemicals in the environment. 1) The model chemical targeted a specific receptor with high potency resulting in the capacity to elicit toxicity at very low exposure levels. 2) Processes that are critical to population sustainability were disrupted as a consequence of the initial chemical:target interaction. 3) Adverse consequences of the initial exposure event persisted into subsequent unexposed generations. The identification of such pathways and the characterization of their susceptibility to disruption by environmental chemicals can significantly refine the hazard risk characterization process.

## Materials and Methods

### Daphnids

Transcription factors were cloned from tissues of *D. pulex* (clone NP6 [15]) since we had previously identified and annotated several transcription factors from this species [19]. Life cycle experiments were performed with *D. magna* (clone NCSU1 [15]) due to the greater fecundity associated with this species. Animals were cultured and used in experiments under rearing conditions described previously [30]. Cultured daphnids were raised in media reconstituted from deionized water [41]. *D. pulex* were maintained at a density of 20 daphnids in 40 ml of media and were fed once daily with 1.4×10^7^ cells of algae (*Pseudokirchneriella subcapitata*) and 0.4 mg (dry weight) Tetrafin™ fish food suspension prepared as described previously [42]. *D. magna* were reared at a density of 40 daphnids in l liter of media and were fed twice daily with 1.4×10^8^ cells of *P. subcapitata* and 4 mg dry weight of fish food suspension. Media was changed 3 times per week. Cultured daphnids were kept in incubators maintained at 20°C with a 16/8 hour light/dark cycle.

### Transcription Factor Cloning

The SV Total RNA Isolation System (Promega) was used to isolate RNA from female *D. pulex*. Oligonucleotide primers were designed to cover the open reading frame of dappuMet, dappuPNR and dappuDSF based on wFleaBase: the Daphnia Genome Database (http://wfleabase.org/). Primer sequences used to amplify the respective cDNAs are provided in Table 1. Amplification of the dappuMet sequence was performed with an iCycler Thermal Cycler (Bio-Rad, Hercules, CA) using 0.25 U Phusion Hot Start DNA Polymerase (New England Biolabs, Ipswich, MA), 5 µl of 5× Phusion GC Buffer, 0.75 µl DMSO, 200 µM dNTP, 0.5 µM primers, 100 ng template cDNA for a total amount of 25 µl. PCR conditions consisted of hot start at 98°C for 30 sec, followed by 40 cycles with each cycle consisting of 10 sec at 98°C, 30 sec at 58°C, and 45 sec at 72°C. Amplification of dappuPNR and dappuDSF were similarly performed but with 2X PCR Mastermix (Promega) at 94°C for 2 min, followed by 40 cycles with each cycle consisting of 30 sec at 94°C, 30 sec at 54.5°C, and 2 min at 72°C. The amplified DNA fragments were cloned into the pCR 4-TOPO vector (Invitrogen, Carlsbad, CA) following the manufacture’s protocol. Plasmid DNA was sequenced by Eurofins MWG Operon (Huntsville, AL). The Met gene from *D. magna* also was cloned (dapmagMet) using procedures as described for dappuMet.

**Table 1:** Oligonucleotide primers used in the PCR amplification of various transcription factors.

Use	Gene	Primer Sequence
cDNA cloning	dappuPNR	forward: 5′-AGTATCCCAACGGAGTGACG-3′
		reverse: 5′-TACTGAGGATCCCGGGTCA-3′
cDNA cloning	dappuDSF	forward: 5′-CATCGTCTCCCCTCCTTGTA-3′
		reverse: 5′-GGGGGAAAGGAAATCTCATC-3′
cDNA cloning	dappu & dapmag Met	forward: 5′-CCTTACGGAAAGCATCTTTAGTG-3′
		reverse: 5′-CGTATGAATTAAAACAGCTTATTAGAAGTC-3′
Reporter Assay	GAL4	forward: 5′- *TATT* **ACTAGT**GGCATGAAGCTACTGTCTTCTATCGAACAAG-3′
		reverse: 5′- *AATT* **TTCGAA**TCTAGATGATATCAACGCGTCAAGTCGAC-3′
Reporter Assay	dappuPNR	forward: 5′-*TACTAT* **GAATTC**CGACCGGAAATTCTGGCCGAA-3′
		reverse: 5′-*TACTAT* **TTCGAA**TTAATTTTTGTACATATCGCAGAG-3′
Reporter Assay	dappuDSF	forward: 5′-*CAAC* **GAATT**CAACAGCGTCCATCACCATTTC-3′
		reverse: 5′-*CTCT* **TTCGAA**CATCGATGAAACCAAACCAA-3′
Reporter Assay	dappuMet	forward: 5′-*TACTAT* **GAATT**CATACATCAGAATGTGGATTTACGGGT-3′
		reverse: 5′-*TACTAT* **ACGCGT**TCACGGACTACTAGTTCCAG-3′

Bold denotes added restriction sites and italics denote spacer nucleotides added to facilitate proper cutting of the sequence. Some primers used in the reporter assay constructs were situated upstream or downstream of the sequence targeted for amplification.

### Luciferase Reporter Gene Assays

Chimeric constructs consisting of the transcription factor and a Gal4 DNA binding domain were prepared for use in luciferase-based transcription reporter assays. DNA encoding the 489 nucleotides of the Gal4 DNA binding domain within the pBIND vector (Promega) was amplified using the oligonucleotide primers described in Table 1. The amplified DNA fragments were digested with SpeI and BstBI and cloned into the PMTB vector (Invitrogen). This construct was designated the PMT-Gal4 vector. DNA encompassing the DEF domain of dappuPNR and dappuDSF and the PAS domains of dappuMet were amplified using oligonucleotide primers depicted in Table 1. Amplified sequences are underlined in the transcription factor nucleotide sequences provided in the Supplementary Information (Figs. S1, S2, and S3). The PCR products were digested with the appropriate enzymes (dappuMet: EcoRI and MluI; dappuDSF and dappuPNR: EcoRI and BstBI) and cloned into the PMT-Gal4 vector. Vector containing the SRC gene (pAC 5.1/V5-His A-FISC), isolated from mosquito (*Aedes aegypti*), was a generous gift from Dr Jinsong Zhu, Department of Biochemistry, Virginia Polytechnic Institute and State University, Blacksburg, VA. The reporter gene vector used in the assay (pGL5-Luc, Promega) contained the luciferase gene with five upstream GAL4 binding sites. The pPAC-β-gal vector, containing the β-galactosidase gene, served as a control for transfection efficiency and was a kind contribution from Dr. Robert Tjian (University of California, Berkeley).

Reporter gene assays were performed in *Drosophila* Schneider (S2) cells (Invitrogen). *Drosophila* S2 cells were grown in Schneider’s medium (Gibco, Carlsbad, CA, USA), containing 10% heat inactivated fetal bovine serum (Gibco), 50 units/ml penicillin G (Fisher Scientific, Pittsburgh, PA), 50 µg/ml streptomycin sulfate (Fisher Scientific) and incubated at 23°C under ambient air atmosphere. Cells were seeded at a density of 3×10^6^ in a 35 mm plate and transfected 16–23 hours after plating when the cells were at 50–70% confluence. Transfections were performed by calcium phosphate DNA precipitation with the relevant plasmids. Following transfection, cells were washed and transcription induced with the addition of CuSO_4_ at a final concentration of 500 µM for 24 hours. Transfected cells were treated with the chemicals for 24 hours with Ex-cellTM 420 insect serum-free medium with L-glutamine (SAFC Biosciences, Sigma, St. Louis, MO) and harvested for luciferase and β-galactosidase determinations. Luciferase activities were measured using the luciferase Assay System (Promega), and normalized to β-galactosidase activities which were measured by the β-galactosidase Enzyme Assay System with Reporter Lysis Buffer (Promega), according to the manufacturer’s recommendation. Each experiment was repeated at least three times.

Compounds evaluated in the transcription reporter assays were: methyl farnesoate (95%, Echelon Biosciences Inc., Salt Lake City, Utah), pyriproxyfen (99.5%, Chem Service, West Chester, PA), methoprene (99%, Chem Service) and kinoprene (96%, Chem Service). Chemicals were dissolved in DMSO for reporter assays at a final assay concentration of 0.050%, v/v.

### Male Sex Determination

The potency of several juvenoid analogs in stimulating male sex determination of daphnids was determined generally as described previously [34]. Compounds used were the same as used in the transcription reporter assays. All test compounds were dissolved in ethanol with a final ethanol concentration in treatments and controls of 0.050%, v/v. Female daphnids, carrying embryos in their brood chambers, were selected from the cultures and placed individually in 50-ml beakers containing 40 ml media and the desired concentration of juvenoid analog. Test solutions were changed daily and daphnids were observed daily for the release of broods of offspring. Food was provided to each beaker as 7×10^6^ cells of algae (*P. subcapitata)* and 0.20 mg (dry wt) of fish food homogenate [42] daily. Treatments were replicated 10-times (ie., one animal per beaker, 10 beakers per treatment). Assays were terminated when all maternal daphnids in the experiment had released their second brood of offspring.

The number of offspring present in the second brood released by each maternal daphnid was quantified and sex of individual daphnids within that brood was determined. Sex of individual offspring was established microscopically with males being discerned from females by the longer first antennae [14]. Daphnids typically produce only female offspring under these culture and assay conditions in the absence of juvenoid compound.

### Life Cycle Assessment

Daphnids (*D. magna*) were exposed to concentrations of pyriproxyfen over their life cycle to test the hypothesis that maternal exposure to this methyl farnesoate mimic causes transgenerational effects. Individual female daphnids were exposed to a series of tightly spaced dilutions of pyriproxyfen for 21 days during which time effects on parental survival, growth, and molt cycle duration was evaluated. In addition, effects of pyriproxyfen on brood size and sex ratio of offspring was determined. Results were compared to those derived from 10 control organisms that were exposed only to the solvent used to deliver pyriproxyfen (ethanol, 0.020%, v/v). Animals were exposed individually in 50 ml beakers containing 40 ml of media. Solutions were exchanged every 2–3 days. Test beakers were provided 3.5×10^6^ cells of algae (*P. subcapitata)* and 0.10 mg (dry wt) of fish food homogenate [42] twice daily for daphnnids <7 days old and twice these amounts, for animals >7 days old. Experiments were maintained in incubators at 20°C and a light:dark photoperiod of 16∶8 hr. This experimental design has been described in detail previously [43].

Experimental animals were examined daily for survival, ecdysis, and offspring production. Exuvia and offspring were removed from the beakers when observed and sex of individual offspring was determined microscopically based upon the length of the first antennae [14]. At 21 days exposure, length of individual parental organisms was determined as the distance from the top of the helmet to the base of the shell spine.

One female offspring derived from a mixed (males and females) brood from each of 10 maternal daphnids exposed to 0.22 nM pyriproxyfen were raised to reproductive maturity in the absence of pyriproxyfen. Ten offspring from unexposed daphnids were similarly isolated and raised to reproductive maturity. Survival and length of these organisms, size of their first brood of offspring and sex of individuals within the first brood produced by these organisms were determined as additional indicators of transgenerational effects of pyriproxyfen.

### Statistics and Modeling

Significant differences between treatment and controls were evaluated using Student’s t test at p = 0.05. All concentration-response curves were generated using the logistic equation. Statistics and curve generation were performed using Origin software (OriginLab Corp., Northampton, MA). The amino acid sequences were deduced from the nucleotide sequences using ExPASy software (http://www.expasy.org/). Amino acid sequence alignments were performed using ClustalW (http://www.genome.jp/tools/clustalw/).

## Supporting Information

Figure S1 **Open reading frame nucleotide sequence of the of the dappuPNR cDNA.** Underlined sequence denotes the portion that was used in the transcription reporter assays.
(TIF)

Figure S2 **Open reading frame nucleotide sequence of the of the dappuDSF cDNA.** Underlined sequence denotes that which was used in transcription reporter assays.
(TIF)

Figure S3 **Open reading frame nucleotide sequence of the of the dappuMet cDNA.** Underlined sequence denotes that which was used in transcription reporter assays.
(TIF)

Figure S4 **Open reading frame nucleotide sequence of the of the dapmagMet cDNA.**
(TIF)
