# Supplementary figures and images for: A Transgenerational Endocrine Signaling Pathway in Crustacea
Source: PLoS One. 2013 Apr 17;8(4):e61715. doi: 10.1371/journal.pone.0061715 (PMC3629115; doi:10.1371/journal.pone.0061715)

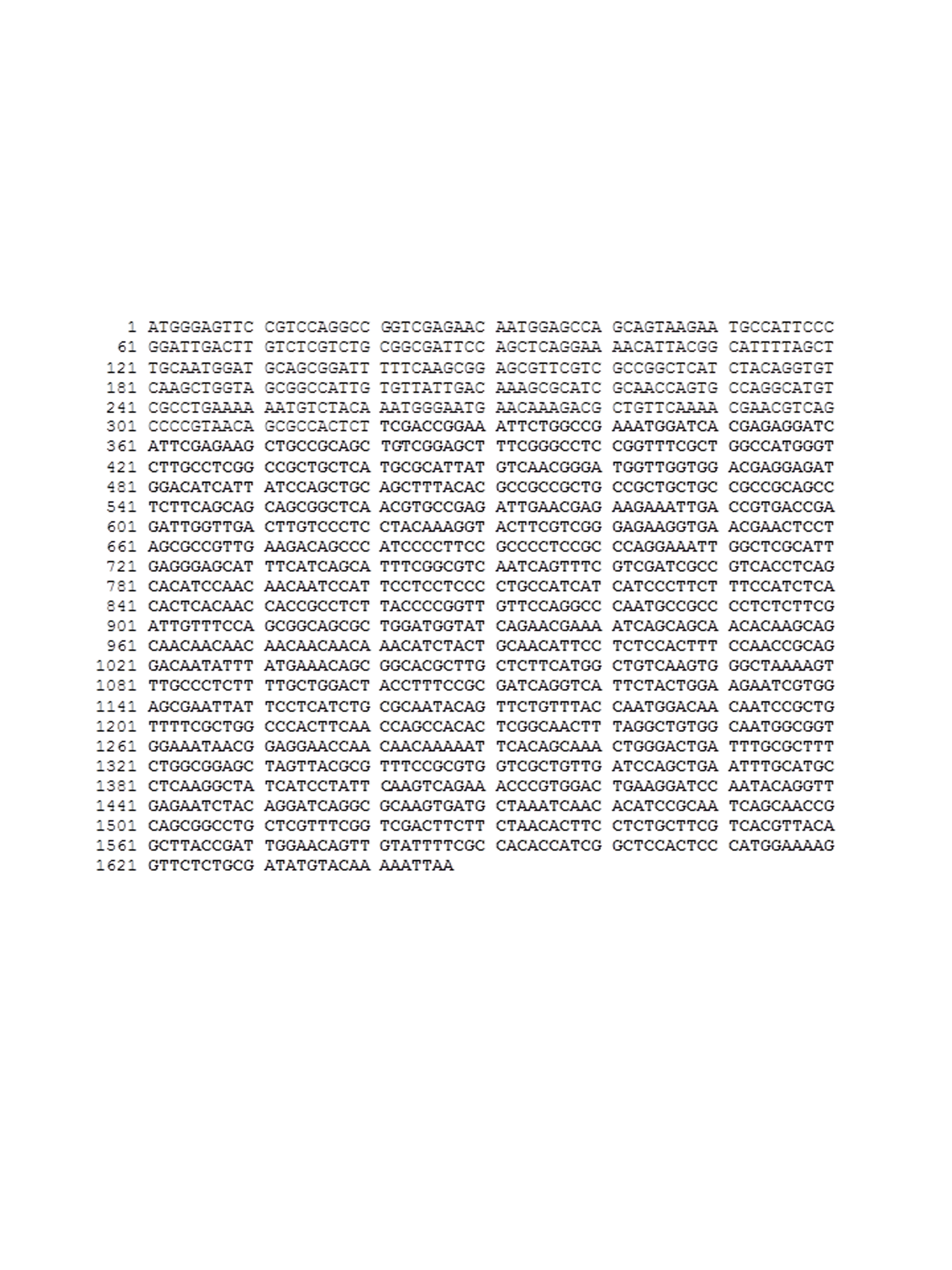

Supplement: Figure S1 — Open reading frame nucleotide sequence of the of the dappuPNR cDNA. Underlined sequence denotes the portion that was used in the transcription reporter assays. (TIF) [file pone.0061715.s001.tif]

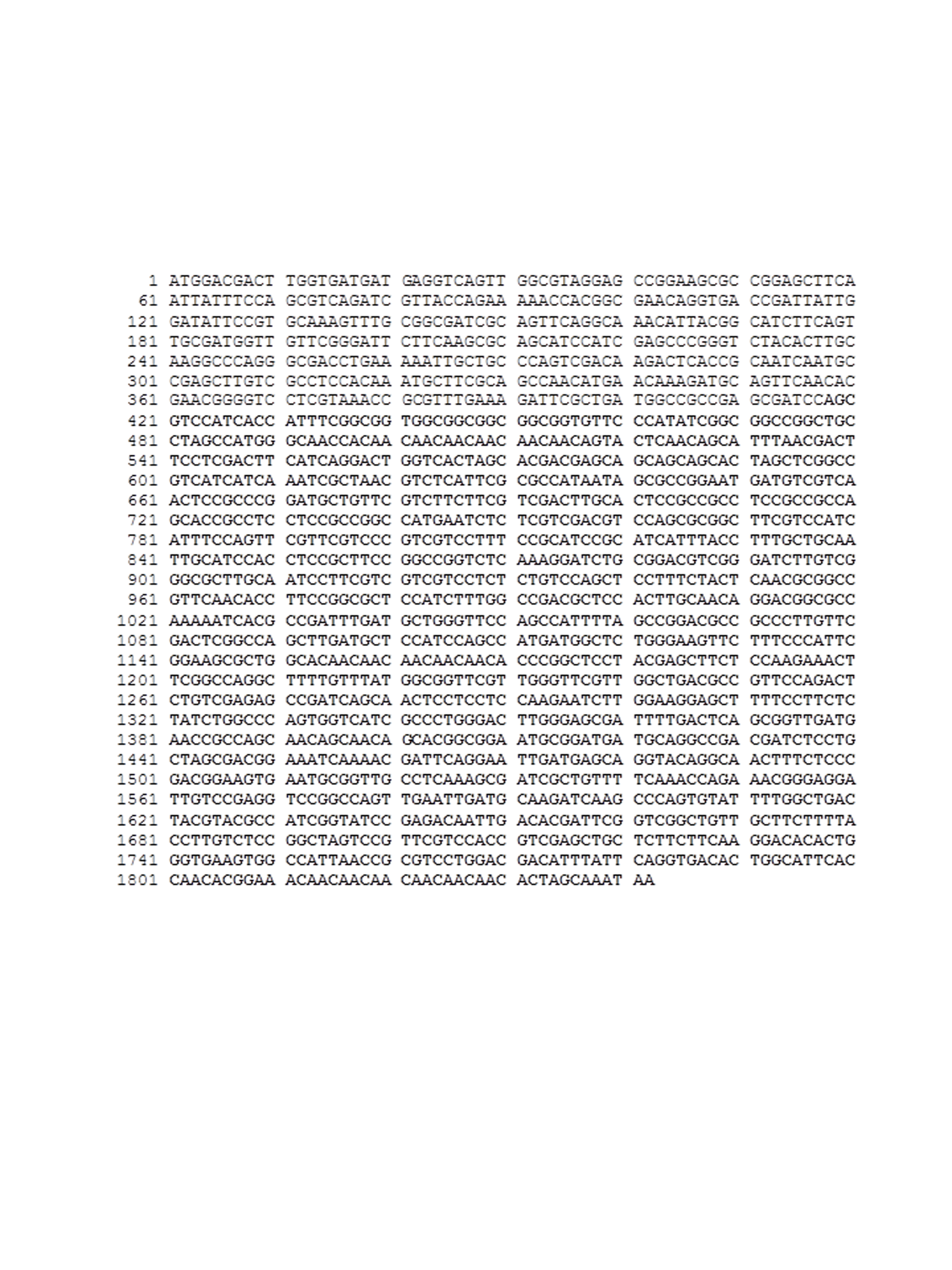

Supplement: Figure S2 — Open reading frame nucleotide sequence of the of the dappuDSF cDNA. Underlined sequence denotes that which was used in transcription reporter assays. (TIF) [file pone.0061715.s002.tif]

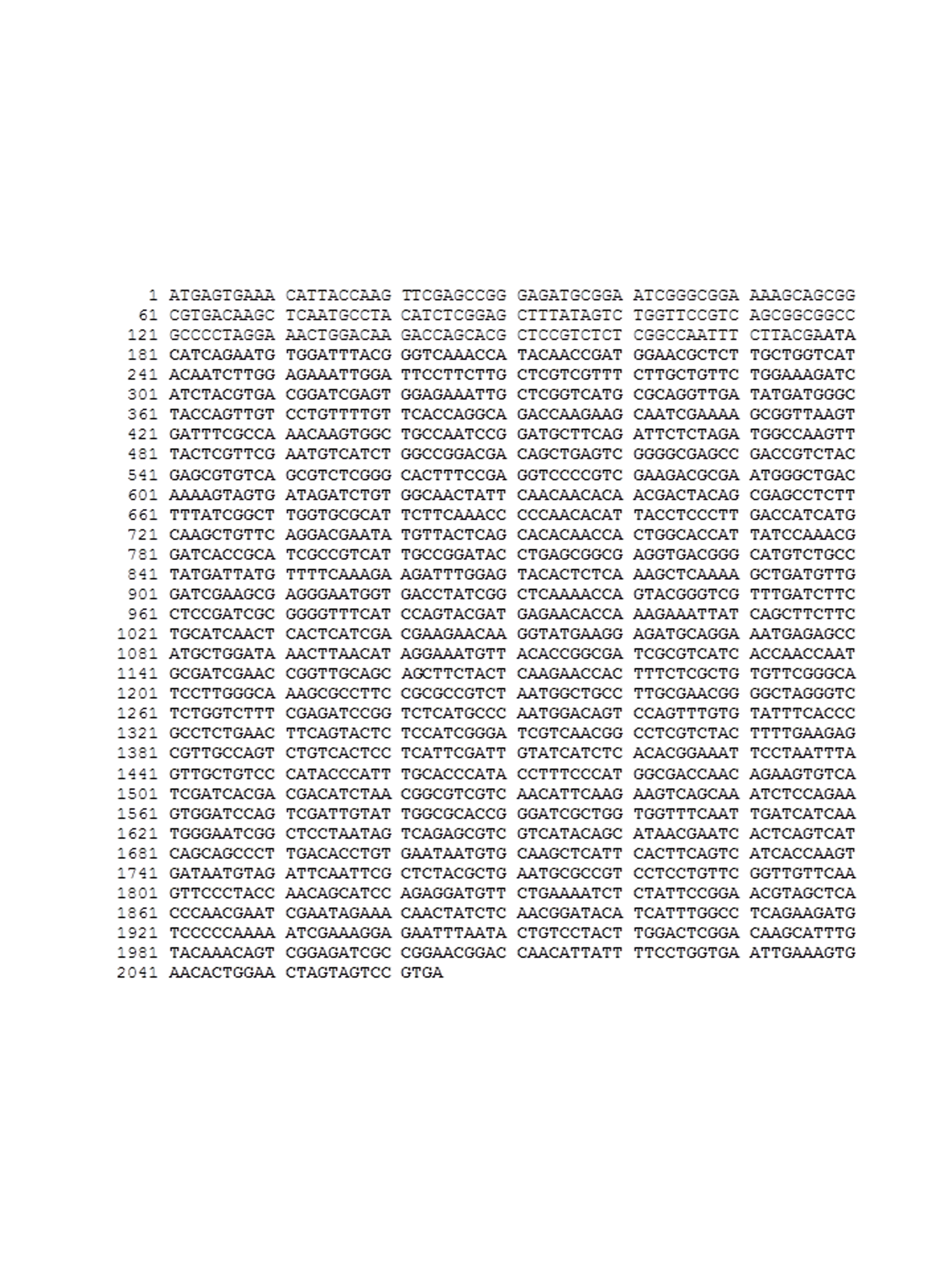

Supplement: Figure S3 — Open reading frame nucleotide sequence of the of the dappuMet cDNA. Underlined sequence denotes that which was used in transcription reporter assays. (TIF) [file pone.0061715.s003.tif]

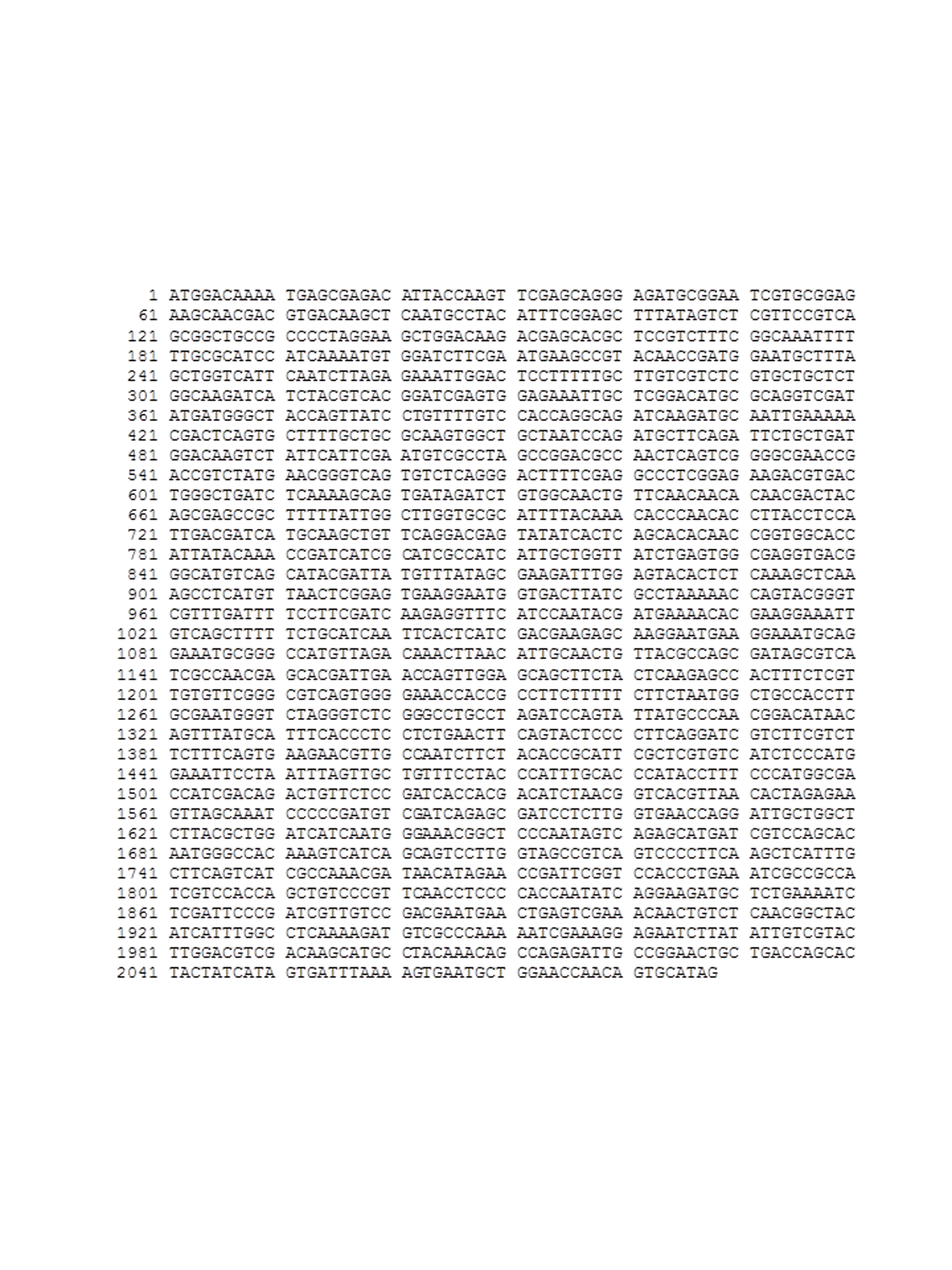

Supplement: Figure S4 — Open reading frame nucleotide sequence of the of the dapmagMet cDNA. (TIF) [file pone.0061715.s004.tif]
